# Special Issue: The Impact of Early Life Nutrition on Gut Maturation and Later Life Gut Health

**DOI:** 10.3390/nu15061498

**Published:** 2023-03-21

**Authors:** Myrtani Pieri, Vicky Nicolaidou, Christos Papaneophytou

**Affiliations:** Department of Life Sciences, School of Life and Health Sciences, University of Nicosia, Nicosia 2417, Cyprus; pieri.m@unic.ac.cy (M.P.); nicolaidou.v@unic.ac.cy (V.N.)

Nutrition during early life plays a crucial role in determining a child’s long-term health [1]. At the same time, it significantly contributes to gut maturation and overall gut health later in life [2]. It is well established that the first 1000 days of life, which start from conception and end at the beginning of the third postnatal year, are essential to overall health later on [3]. A child’s neurological development is most active during this period, making it a critical window for proper nutrition [4]. Furthermore, evidence suggests that a child’s susceptibility to developing chronic noncommunicable diseases later in life may also be influenced by their nutrition during the first 1000 days [5]. Therefore, ensuring adequate and appropriate nutrition during this period can have long-lasting health benefits [6]. Notably, the neonatal period is vital for the development and maturation of the gut microbiome, which can have a lasting impact on health outcomes [7,8]. During this period, the gut microbiome plays a critical role in protecting against enteric infections and is essential for the adequate development of the immune system [9].

The gastrointestinal tract (GIT) of humans represents an incredibly complex ecosystem, characterized by a diverse assemblage of microbial residents. These microorganisms have established a complex relationship with their host over an extended period, resulting in co-evolution [10]. The entirety of the gut microbiota’s genetic repertoire has been estimated to contain roughly 3.3 million microbial genes [11]. The dynamic interplay between various factors such as genetics, dietary habits, and environmental factors shapes this intricate ecosystem. Consequently, maintaining a stable and healthy gut microbiome is crucial for human health, particularly in the early stages of life [12].

The gut microbiome undergoes several changes during the first year of life [13]. Various factors, including mode of delivery, feeding practices, and the introduction of solid foods, influence these changes. Furthermore, nutritional deficiencies during the early stages of life can result in an immature gut microbiome and an increased risk of gut-related issues [14].

Interventions or exposures that could affect the transmission of microbes from the mother to the infant, or the communities themselves, such as changes in the infant’s diet, antibiotic exposure, and cesarean section delivery, are thought to perturb the optimal infant intestinal microbiome [15,16,17].

In contrast, an adequate intake of nutrients, such as protein, carbohydrates, and fats, can lead to proper gut development and function [2]. As mentioned above, the gut microbiome plays a critical role in the development and function of the immune system, nutrient metabolism, and the maintenance of gut barrier function. Notably, the first year of life is crucial for developing the gut microbiome and training the immune system. Bacteria residing in the neonatal gut have garnered significant attention in microbiome research due to their critical role. The initial substantial encounter with these microorganisms occurs during birth, whereupon exiting the womb, microbes colonize the human body and establish themselves in the gut [18]. Dysbiosis, or an imbalance in the gut microbiome, has been linked to numerous health conditions, including obesity, diabetes, allergy and autoimmune diseases. Therefore, understanding how early life nutrition affects the development of the gut microbiome is essential for promoting optimal gut health [19].

It is well known that breastfeeding positively impacts gut maturation and overall gut health [20]. The World Health Organization (WHO) recommends that breastfeeding should be the primary source of infant nutrition [21]. Exclusive breastfeeding for the first six months of life is optimal for a baby’s growth and development. Breast milk contains all the nutrients an infant needs and provides additional benefits, such as protection against infections and chronic diseases [22]. It has also been demonstrated that breastfeeding promotes the growth of beneficial bacteria, such as *Bifidobacteria*, and reduces the abundance of potentially harmful bacteria [23]. This effect is thought to be due to prebiotic oligosaccharides in breast milk, which serves as a food source for beneficial bacteria. In contrast, formula feeding has been associated with a less diverse gut microbiome and an increased abundance of potentially harmful bacteria [24].

The process of gut maturation in the early stages of life entails permanent structural and functional modifications that reach their peak during weaning. This transition period involves the replacement of maternal milk with solid (complex) food [25]. The timing of the introduction of solid foods is also an essential factor in gut microbiome development. The early introduction of potentially allergenic foods, such as peanuts and eggs, has been shown to reduce the risk of developing food allergies. This effect is thought to be due to the promotion of immune tolerance through early exposure to these foods. Additionally, the introduction of solid foods has been associated with a shift in the gut microbiome composition, with a decrease in beneficial bacteria and an increase in potentially harmful bacteria [25].

Several interventions have been developed to promote optimal gut health during early life (reviewed in [2]). These interventions include the use of probiotics, prebiotics, and synbiotics. Probiotics are live bacteria that can be added to formula or given as a supplement, intending to promote the growth of beneficial bacteria. Prebiotics are non-digestible fibers that serve as a food source for beneficial bacteria. Synbiotics combine probiotics and prebiotics to facilitate the growth of beneficial bacteria. Furthermore, numerous studies have evaluated the effectiveness of these interventions in promoting optimal gut health. However, results have been mixed, with some studies showing a positive effect and others showing no effect. Therefore, further research is needed to determine the optimal timing, dosage, and combination of probiotics, prebiotics, and synbiotics to promote optimal gut health (reviewed in [26]).

Together, the above data highlight the effect of early-life nutrition on gut microbiome development and maturation. Therefore, it is vital to prioritize appropriate nutrition during early life to ensure the gut microbiome develops properly, promoting optimal health and well-being. Breastfeeding and the early introduction of potentially allergenic foods have positively impacted gut health. In addition, clinical interventions, such as probiotics, prebiotics, and synbiotics, have been developed to promote optimal gut health. While these interventions show promise, further research is needed to fully understand their effectiveness and determine the most appropriate use and dosages.

The studies featured in this Special Issue of *Nutrients* aim to provide a qualified and evidence-based discussion on the impact of early-life nutrition on gut maturation and later-life gut health. That will offer valuable information for scientists involved in experimental and clinical research, as well as suggestions and recommendations for how to regulate gut maturation and induce the process precociously or delay it through environmental and dietary intervention.
